# The free-living ciliate *Tetrahymena pyriformis* inactivates engulfed influenza A(H1N1)pdm09 virus via two distinct types of endosomes

**DOI:** 10.1038/s41598-025-19490-w

**Published:** 2025-10-15

**Authors:** Valentina I. Pushkareva, Anastasia S. Krepkaia, Anna V. Ignatieva, Natalya V. Shevlyagina, Svetlana G. Andreevskaya, Elizaveta Fofanova, Elena V. Sysolyatina, Vladimir G. Zhukhovitsky, Elena I. Burtseva, Svetlana A. Ermolaeva

**Affiliations:** 1 The Gamaleya Research Institute of Epidemiology and Microbiology of the Ministry of Health of the Russian Federation, Moscow, Russia; 2 Department of Comparative and Developmental Physiology, Koltzov Institute of Developmental Biology RAS, Moscow, Russia; 3 Russian Medical Academy of Continuing Professional Education (RMANPO), Ministry of Public Health, Moscow, Russia

**Keywords:** Ciliate *Tetrahymena pyriformis*, Influenza A, Inactivation of influenza virus, Protists, Endosome, Influenza virus, Viral epidemiology

## Abstract

Wild aquatic birds are a major reservoir of the influenza A virus in natural ecosystems, facilitating its entry into the aquatic microbial food web through their feces. Free-living protozoa and particularly bacterivorous ciliates are essential players of the microbial food web. This study investigates the interactions between the Influenza A(H1N1)pdm09 virus and the ciliated protozoan *Tetrahymena pyriformis* at the population and ultrastructural levels. Co-cultivation of Influenza A(H1N1)pdm09 and *T. pyriformis* resulted in a decline and eventual complete elimination of the viral population. The inactivation of the virus was not mediated by products excreted by T. pyriformis but required A(H1N1)pdm09 endocytosis. Viruses ingested by protozoa lost their virulence within 48 hours post infection (hpi) and, as determined by hemagglutination assays, were entirely inactivated within 72 hpi. When lysates infected with A(H1N1)pdm09 *T. pyriformis* were applied to MDCK cells 1.5 and 24 hpi the undamaged part of ingested virions caused a cytopathic effect. Confocal laser scanning microscopy (CLSM) and transmission electron microscopy (TEM) of infected *T. pyriformis* cells revealed large food vacuoles, including multiple undamaged and partly processed virus particles, at 1.5 and 24 hpi. Furthermore, TEM identified coated and half-coated small one-virus endosomes that predominated at 48 hpi. These results demonstrated that A(H1N1)pdm09 inactivation by *T. pyriformis* includes two types of endosomes that dominated at different periods of interpopulation interactions. The process of A(H1N1)pdm09 inactivation in protozoan cells occurs rapidly, but not instantaneously, that suggesting a dual role of protozoa in the fate of influenza A viruses in natural ecosystems, both as predators and as potential vectors.

## Introduction

Influenza A virus is widespread in nature, and aquatic birds, which is known to have a wide range of susceptible hosts, including birds, some mammals, and humans. Wild aquatic birds, are considered a major reservoir of influenza A virus subtypes in the natural ecosystem, they support virus multiplication and contribute the greatest diversity of its population and evolution^28,56,59^. The number of influenza virions entering the environment in the fecal matter of each infected duck is evaluated as approximately 10^10^ 50% embryonic infectious dose (EID_50_) ^57^. Further, avian influenza viruses are stable in water^50,56^ and have been isolated from the surface of ponds containing a large number of waterfowl^17,18^. Recent reports indicate that the avian influenza A virus can retain infectious activity in water for up to 7 months^42^. Although aerosol transmission cannot be dismissed, the larger number of positive cloacal than tracheal swabs, the high fecal virus titer, and the stability of the virions in water suggest that low pathogenic avian influenza viruses persist in duck populations through fecal-oral transmission^56^. This mechanism could partially explain the higher prevalence of infection in surface-feeding (dabbling) ducks than in diving ducks that typically feed in deeper water^22^.

Viruses entering natural aquatic reservoirs through bird feces infiltrate intricate microbial food web. Free-living protozoa represent a significant part of the plankton biomass of the aquatic ecosystems and an essential element of the microbial food web, acting as predators of bacteria and smaller microorganisms while serving as prey for carnivorous protists and metazoans^19^,Bulannga et al., 9,30,41,60. Intensive reproduction, short generation time, high population size, long-term preservation in dormant forms (cysts), and bacteriovourus and/or carnivourus nature of nutrition determine the role of protozoa in interactions with other members of the microbial food web^7,12^. On the other hand, free-living protozoa are a typical prey of not only crustaceans and other multicellular representatives of zooplankton, but as well wild birds and mammals whose diet is based on water filtration^7,15,39^. This intermediate position makes protozoa able to pass smaller microorganisms including pathogens to vertebrates via food chains^13,15,40^.

Interactions between protozoa and viruses are relatively underexplored. Ciliates, primarily *T. Pyriformis*, have been intermittently studied over the past 70 years for possible associations with viruses of humans, and less frequently with viruses of fish, with intriguing but not always definitive results. Subsequently, laboratory investigations were done, commonly with Tetrahymena. The viruses included influenza virus, encephalomyocarditis (EMC) virus, measles virus vaccinia virus, coxsackie B-5 virus, adenovirus type 2 and type 3, poliovirus and Simian rotavirus SA11 (Groupé and ^14,25,48,53^, Kling, ^24^, Kim and Unno et al., ^6,23,34^). While there is evidences that ciliates destroyed or inactivated some viruses of mammals, Tetrahymena spp were shown to increase the titer of the chum salmon reovirus upon incubation of ciliates and CSV at 22 °C^37^

Rates of protozoan grazing depend on virus although mechanisms determining the rates are unknown^19,35^. Experimental conditions are important for virus inactivation: a number of studies on interactions between the T4 bacteriophage, phages Phi X174 and MS2 and the ciliate Tetrahymena spp showed alternative results concerning phage inactivation although all studies demonstrated T4 ingestion by the protozoan^2,16,38^. For human viruses, the results of their interactions with protozoa range from accumulation on the protozoan surface to rapid elimination, depending on unidentified factors^5,35^. Mechanisms that control interactions between protozoa and viruses as well as a potential role of protozoa in circulation of human viruses in aquatic ecosystems are essentially unexplored^5,16,19,35,38^. In this work, we used the ciliate *T. pyriformis* as a model organism to investigate interactions between protozoa and the human Influenza A(H1N1)pdm09 virus.

The significance of this study is in characterization of interactions between influenza A virus and *Tetrahymena pyriformis*, a model protist representing free-living protozoa. The results will enhance our understanding a fate of Influenza A virus in aquatic ecosystems and underly a role of the microbial food web in viral maintenance in natural ecosystems. Influenza A virus is prototypic among viral infections with natural foci that causes infections of both anthropogenic and zoonotic origin, leading to sustained transmission and the emergence of novel viruses^4,10,28,52^.

## Materials and methods

### Cultivation of *Tetrahymena pyriformis*

The *T. pyriformis* strain GL was obtained from Institute of Cytology, Russian Academy of Sciences, St-Petersburg, Russia. *T. pyriformis* was routinely maintained axenically at a temperature of 28 °C in the brain heart infusion (BHI) broth (BD, Franklin Lakes, NJ, USA) diluted 1:10^40^. Other workers have reported the growth of Tetrahymena in culture media that had been designed for the mammalian cells^37^. To optimize conditions for the protozoan-viral association, the protozoa were adapted to the Eagle’s MEM medium (Eagle’s MEM, Paneco Ltd, Moscow, Russia) supplemented with a double set of amino acids (Biolot Ltd, Sankt-Petersburg, Russia) by cultivation in this medium for 7 days. The protozoan density utilized in the experiments was 10^5^ trophozoides per ml.

### Cell line management

MDCK cells (Madin-Darby canine kidney cells (NBL-2), ATCC CCL-34) was obtained from Central of Disease and Control (CDC). Cell line was cultivated using standard method^21,58^ in the Eagle’s MEM medium supplemented with a double set of amino acids with 7 % fetal bovine serum (BioSera), 50 μg/ml gentamicin, at 37°C, 5% CO_2_ atmosphere.

### Virus propagation

Used in the study Influenza A/Guangdong Maonan/SWL1536/2019 H1N1pdm09 (A(H1N1)pdm09 virus was a courtesy of the Francis Crick Institute (London, UK) in collaboration with the Global Influenza Surveillance and Response System (GISRS), which passed 4 passages in chicken embryos. Routinely, A(H1N1)pdm09 was propogated in a culture of MDCK cells at a temperature of 37 °C for 48 hours^21,58^ with 1μg/ml trypsin TPCK-treated (tosyl phenylalanyl chloromethyl ketone). Then the virus-containing liquid was filtered using a 0.22 μm syringe filter (TPP, 0.22 μm, PES, 33 mm, gamma-sterilized), aliquoted and stored at -70°C.

### Modeling of the A(H1N1)pdm09 / T. pyriformis association

The experimental design was developed based on the works of the Groupé^14^ and the Olive^35^ with some modifications. An A(H1N1)pdm09 suspension in 10 ml Eagle’s MEM medium with an hemagglutination titer of 128 hemagglutination units per 50 µl (HAU/50 µl) and an infectious titer of 10^7.75^TCID_50_/0,1 ml (50% tissue culture infectious dose) was added to 10 ml of a *T. pyriformis* suspension with the concentration of 10^5^ cells/ml. The mixed culture was incubated at a temperature of 25 °C for 7 days. 0.5 ml aliquots were taken at various time intervals (1.5 h, 24 h, 48 h, 72 h, 96 h, 120 h, 168 h). Taken aliquots were centrifuged at 1000 rpm for 5 minutes, the supernatant was passed into a sterile tube to be frozen, the pellet was washed with 500 µl of the Eagle’s MEM medium, then the pellet was resuspended in 250 µl of the MEM medium, then the samples (the supernatant and the cell sediment suspension) were frozen and stored at −70 °C. As a virus control (positive control), the A(H1N1)pdm09 suspension was diluted with the equal volume of the sterile Eagle’s MEM medium and incubated under the same conditions and aliquots were taken at the same time points and processed in the same way. An intact culture of *T. pyriformis* grown in the Eagle’s MEM was used as a negative control.

### A(H1N1)pdm09 growth in the medium conditioned by T. pyriformis

*T. pyriformis* was grown in to the Eagle’s MEM medium supplemented with a double set of amino acids for 5 days. Then the supernatant was sterilized via nitrocellulose filter (1.0 μm). The *A(H1N1)pdm09* was mixed with the conditioned medium in the ratio 1:1 (v:v). As a control, fresh medium was added to the virus culture in the same ratio.

### Hemagglutination (HA) assay

The hemagglutination assay was performed by standard technique^58^. 50 μl of 0.75% suspension of human erythrocytes of the 0(I) blood group, diluted with 0.9% PBS (phosphate-buffered saline, pH 7.2) were added to 50 μl of serial two-fold dilutions of *T. pyriformis*/ A(H1N1)pdm09 suspension and placed in round-bottom 96-well plates. The plates were incubated 60 minutes at a temperature of +6 °C until complete erythrocyte sedimentation. The titer of the virus was expressed in HAU/50 µl, corresponding to the reciprocal of the last dilution, giving erythrocyte agglutination^49^. The hemagglutination activities of the positive (A(H1N1)pdm09 alone) and negative (*T. pyriformis* alone) cultures were evaluated in the same way.

### Reverse transcription/ quantitative PCR (RT/Q-PCR)

Viral RNA was isolated using the QIAamp Viral RNA Mini kit (Qiagen GmbH, Hilden, Germany), followed by amplification using the CDC rRT-PCR Flu Panel test system^58^ and the CFX96 PCR amplifier (BioRad, Hercules, CA, USA) for the qualitative determination of influenza A H1N1 virus^45^ with using a calibrator for quantitative analysis. Results were shown as a mean of genome equivalent of influenza virus per ml (GE/ml). The detectable limit CDC rRT-PCR Flu Panel test system was 1000 GE/ml. GE/ml value calculated from three independent experiments. The concentration of genome equivalent of influenza virus was determined with using standard curve (R^2^=0.999).

### The tissue culture infectious dose (TCID_50_) assay

Infectious titers of A(H1N1)pdm09 were established in the MDCK cell culture as follows a confluent MDCK cell culture monolayer was formed in 96-well plates^58^. Then serial decimal sample dilutions were added to the MDCK monolayer washed twice with MEM Double Amino Acid Kit Eagle’s Medium (Biolot). Infected cells were incubated at 37°C and 5% CO_2_ for 1 hour, then 100 μl of Eagle’s medium MEM supplemented with a double set of amino acids, 50 μg/ml of gentamicin, and 2 μg/ml of trypsin (TPCK), was added to all wells, and cells were incubated at 37°C and 5% CO_2_ for 48 hours. Cytopathic effect (CPE) was evaluated visually using an Olympus CKX31 microscope. Infectious virus titer (lg(TCID_50_)) was calculated using the Spearman-Kerber formula^27^ .

### Fluorescent microscopy

Samples placed in the 96-well plates and fixed with a 4% paraformaldehyde were treated with 0.5% Triton X -100 and washed with PBS 2 times. Then 50 µl specific antibodies to the influenza A nucleoprotein conjugated with the fluorescent protein GFP (1/16 dilution, PPDP, Saint-Petersburg, Russia) were added, plates were incubated at room temperature for 30 minutes, washed once PBS and twice with bidistilled water, and dried at room temperature. Virus was visualized with ZEISS AxioVert.A1 (Zeiss, Oberkochen, Germany) fluorescent microscope at a magnification of 900x.

### Confocal laser scanning microscopy (CLSM)

0.5 ml samples were placed in microcentrifuge tubes with a filter (AmiconUltra 100K, Merck, Darmstadt, Germany), centrifuged at 13,000 rpm for 5 min, the filter was washed with 450 µl of MEM Eagle medium with a double set of amino acids 2 times. The concentrated precipitate (from 30 to 50 μl) was shaken, collected, transferred to a glass slide, dried, and fixed with 50 μl of 96% alcohol (the glass was left at room temperature until the alcohol was completely dry). After fixation, slides were treated with 0.5% Triton X-100 for 15 min, then washed with PBS 2 times. Next, 50 µl specific antibodies to the influenza A nucleoprotein conjugated with the fluorescent protein GFP (1/16 dilution) were added, glasses were incubated at room temperature for 30 minutes, washed once with PBS and twice with bidistilled water; slides were then dried at room temperature. Images were taken with a confocal microscope ZEISS Ism 800 at a magnification of 40, with a zoom of 3.4, using immersol 518f immersion oil.

### Transmission electron microscopy

Samples were fixed in the Ito-Karnovsky reagent^20^, then post-fixed in 1% aqueous solution of OsO_4_ and 1% uranyl acetate solution (Serva, Germany) in 0.2 M maleate buffer (Science Services GmbH, Germany). Ultrathin sections were obtained with LKB III ultratome (LKB, Switzerland). Then ultrathin sections were contrasted with 1% alcohol solution of uranyl acetate and 0.3% aqueous solution of lead citrate (SERVA Electrophoresis GmbH, Heideberg, Germany) and analyzed using a JEM-2100Plus transmission electron microscope (JEOL Ltd, Tokyo, Japan) at an accelerating voltage of 160 kV.

### Quantification of mitochondria

Mitochondria were counted quantified using at least 20 independent TEM images taken 1.5 h post addition of the A(H1N1)pdm09 virus or a sterile medium as a control.

### MTT assay

The MTT (3-(4,5-dimethylthiazol-2-yl)-2,5-diphenyltetrazolium bromide) dye was added to virus-fed or control *T. pyriformis* cultures up to the concentration of 10 mg ml^−1^. Samples were incubated in the dark at 25 °C for 3 h or 24 h, then the samples were centrifuged at 6000 rpm for 10 min. The precipitate was dissolved in 200 µl of dimethyl sulfoxide (DMSO) and transferred into the wells of a 96-well plate, 100 µl each, then the optical density was detected on an IMark plate reader (BioRad) at a wavelength of 490 nm. To evaluate the *T. pyriformis* metabolic activity (Zilberg, ^61^), optical density was normalized to the number of protozoan cells in the sample.

### Statistics

All experiments were repeated from three to five times. The mean and standard deviation (SD) values were calculated from the entire data set where applicable. Statistical analysis was performed using one-way ANOVA with the post hoc Tukey’s test. The homogeneity of variance assumption was tested using Levene’s test. Statistical differences were considered significant when the *p* value was <0.05.

## Results

### T. pyriformis inactivates viral population

**Fig. 1:**
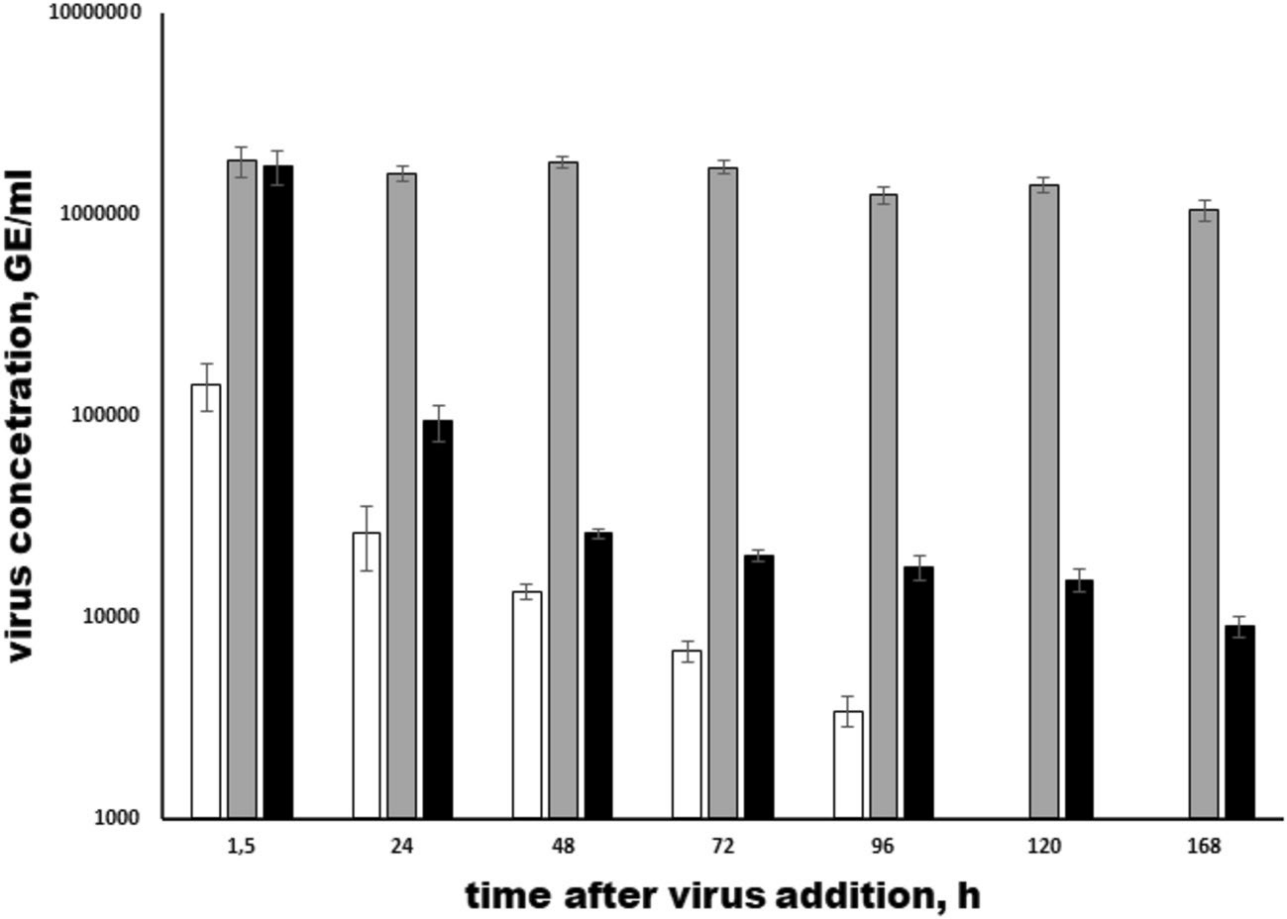
Quantification of A(H1N1)pdm09 RNA in cell lysates and culture supernatants obtained from the A(H1N1)pdm09/ *T. pyriformis* co-culture. The A(H1N1)pdm09 virus and *T. pyriformis* were co-cultivated at 25°C and 0.5 ml samples were collected at specified time points. Reverse transcription followed by Q-PCR was conducted on viral RNA isolated from *T. pyriformis* cell lysates (white) and culture supernatants (black). The unmixed A(H1N1)pdm09 culture was used as a control (grey). The mean virus concentration was genome equivalent per ml (GE/ml) and Standard Deviation (SD) from 3 independent experiments are shown. The detectable limit was 1000 GE/ml; *p*<0.05 compared to the1.5 h time point.

The first objective of our study was to investigate the dynamic of A(H1N1)pdm09 population following its interaction with *T. pyriformis cultures*. The starved protozoan culture was mixed with A(H1N1)pdm09 viruses with multiplicity of infection MOI 1:10^4^ (cell : virus). At different time points, *T. pyriformis* cells were settled down by centrifugation and viral RNA loads was enumerated by RT/Q-PCR reaction separately in cell lysates and in cell-free culture supernatant (Fig. 1). Viral RNA concentration was detected in *T. pyriformis* cell lysates up to 96 hours post infection (hpi), decreasing genome equivalent (GE/ml) from the value of 1.4∙10^5^ GE/ml at 1.5 hpi up to 3.4∙10^3^ GE/ml at 96 hpi. At latter time points, viral RNA was below the detection limits. In culture supernatant, viral RNA loads gradually decreased from 1.7∙10^6^ GE/ml at 1.5 hpi up to 8.9∙10^3^ GE/ml at 168 hpi. The control A(H1N1)pdm09 culture changed insignificantly from 1.8∙10^6^ GE/ml at 1.5 hpi up to 1∙10^6^ GE/ml at 168 hpi, which is typical for cell-grown influenza virus of A(H1N1)pdm09 clade 6B.1A5.5a1. Notably, the viral RNA concentration predominates in the supernatant when compared to the lysate at the different time points. Since the protozoa not only engulf and digest viral particles, but also release their degradation products into the culture medium, the viral RNA from both intact and degraded influenza viruses is more prevalent in the supernatant than in the cell lysate.

**Fig. 2:**
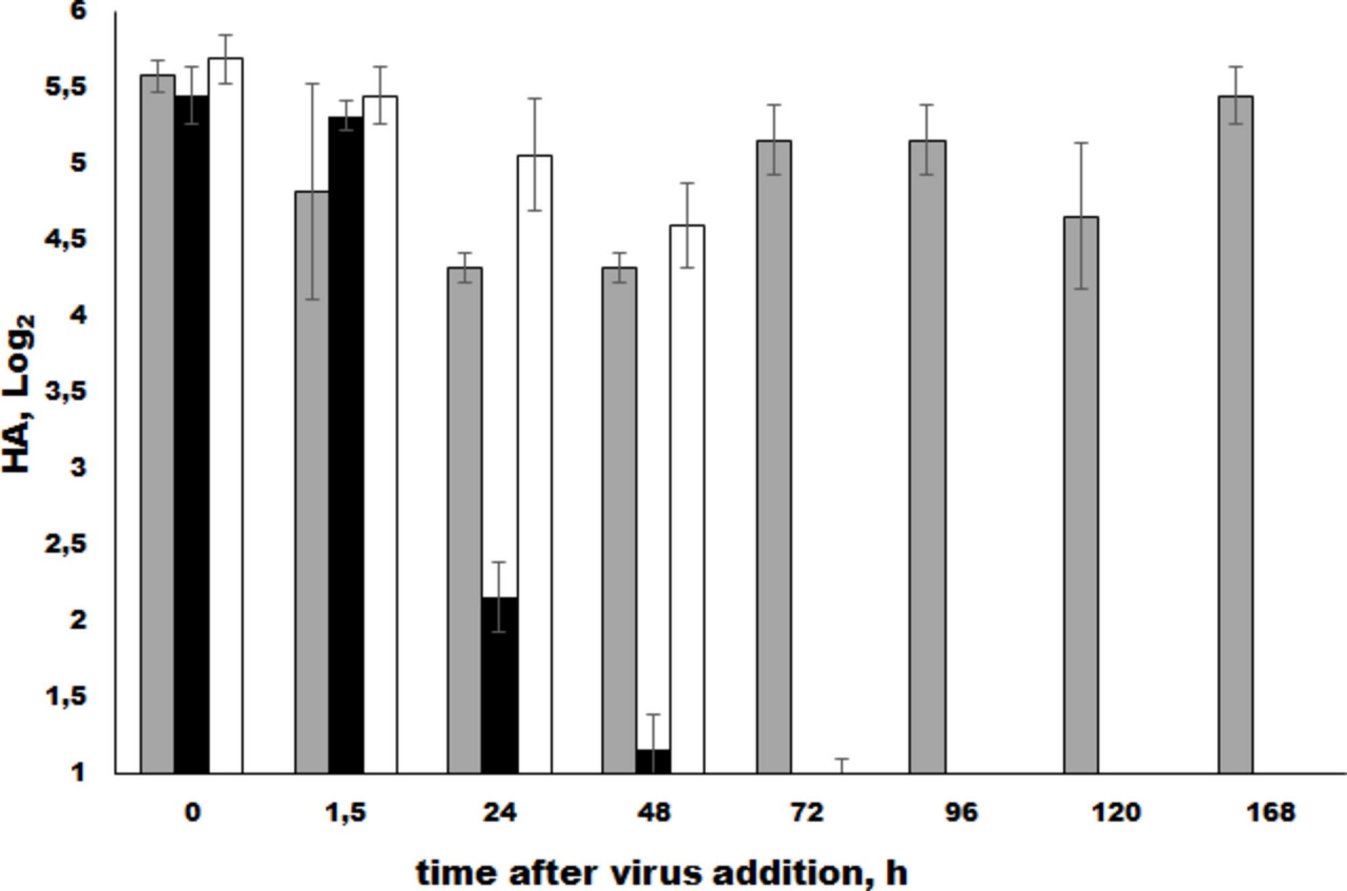
Hemagglutination assay in cell lysates and culture supernatants obtained from the influenza virus A(H1N1)pdm09/ *T. pyriformis* co-culture. Experimental conditions for co-cultivation of A(H1N1)pdm09 virus and *T. pyriformis* are detailed at the Fig. 1 legend. The HA test was performed on *T. pyriformis* cell lysates (white) and culture supernatants (black). The unmixed A(H1N1)pdm09 culture was used as a control (grey). The mean values and Standard Deviation (SD) from 3 independent experiments are shown. *, *p*<0.05 compared to the 1.5 h time point.

To assess whether RNA loads correlated with persistence of viral proteins activity, specifically hemagglutinin, we performed a hemagglutination (HA) assay (Fig. 2). Indeed, these methods exhibit different sensitivities and offer distinct insights into the intracellular processes of the protozoa and the surrounding culture medium. The HA assay confirmed accumulation of the virions in association with protozoan cells within the first hours of interaction. Protozoan lysate-associated signal was detected at nearly the same value up to 48 hpi and then it gradually decreased. The HA titer decreases at 48 hours, suggesting that the HA protein is likely inactivated due to both proteolytic enzymes and low pH levels within the protozoan phagosomes. After 72 hpi, the HA assay was totally negative. HA titers in the cell-free supernatant smoothly diminished from first hours to 48 hpi. After 96 hpi, no HA titers were detected neither in cell-free supernatant no in cell lysates, because HA titers dropped below detectable levels at 96 hpi. It should be noted that the HA titers are higher in the lysates then supernatant. In contrast to the viral RNA quantity (Fig. 1), these results suggest more active degradation of viral particles within the phagosomes than in the culture medium (supernatant). It is evident that, in the viral control, there was an initial decrease in titers over 48 hours, followed by an increase in HA titers at 96 and 168 hours as degradation of viral particles escalates. On the other hand, the predominance of HA titers in the lysate, it may indicate for a change in the mechanisms of virus penetration into the *Tetrachymena*.

### Virus destruction occurred via direct protozoan-virus interactions but not due to T. pyriformis secreted products

The rapid and smooth decline in the number of free-floating viruses and the more abrupt decrease observed for virions associated with protozoan cells suggested that free-floating viruses were ingested and destructed by protozoan cells. The alternative explanation suggested that protozoa produced a certain virus destroying substance(s) that could result in a rapid destruction of viruses in the supernatant.

**Fig. 3:**
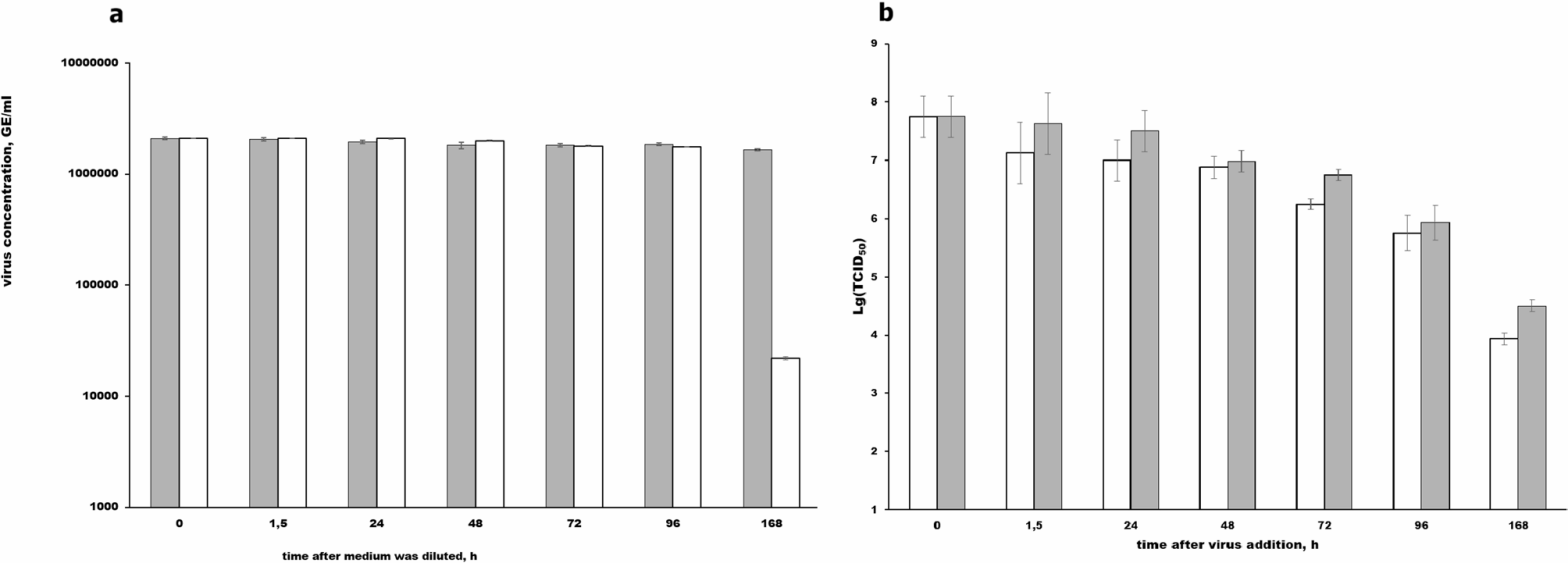
Quantification of influenza virus A(H1N1)pdm09 RNA (**a**) and infectious titers TCID_50_ (**b**) in the conditioned medium. The A(H1N1)pdm09 culture in the Eagles’MEM medium was diluted 1:1 with the culture supernatant of the *T. pyriformis* grown in the same medium for 5 days (white columns) or with the sterile medium (grey columns). The cultures were cultivated at 25°C and 0.5 ml samples were harvested at specified time points for isolate RNA and TCID_50_-assay. Reverse transcription followed by Q-PCR was performed on viral RNA as described previously. The mean virus concentration was genome equivalent per ml (GE/ml) and Standard Deviation (SD) from 3 independent experiments are shown. The detectable limit was 1000 GE/ml, *p*<0.05 comparatively to the initial point. Infectious titers TCID_50_ were determined in the MDCK cells and represented as mean values from 3 independent experiments are shown (*p*<0.05, compared to the initial point).

To choose between these suggestions, we incubated A(H1N1)pdm09 viruses in the medium conditioned by *T. pyriformis* (Fig. 3a,b). Within first 96 hpi, the RT/Q-PCR assay did not reveal noticeable changes of RNA concentrations in the viral culture incubated in the conditioned medium comparatively to the control (Fig. 3a). However, TCID_50_ assay results (Fig. 3b) indicated a gradual decrease in infection titers (log(TCID_50_) over 168 hours of incubation both in the conditioned media and in the control, with a decreased in the conditioned media from 7.75 log(TCID_50_) to 4.5 log(TCID_50_) in control, and from 7.75 log(TCID_50_) to 3.94 log(TCID_50_) in conditioned medium). Comparing with the data on a rapid decreasing of viral loads in the presence of *T. pyriformis* cells, these results suggested that the decrease of viral population in the presence of *T. pyriformis* was due to direct contacts of viruses with protozoa. By 168 hpi, RNA loads in the viral culture incubated with the conditioned medium decreased comparatively with the control (from 1.6∙10^6^ GE/ml to 2.2∙10^4^ GE/ml). We propose that partial virion destruction may have occurred at this time point in both cultures. Such a destruction could make viral RNA accessible for hydrolases that presented in the conditioned medium but not in the control. Results from TCID_50_ assay (see below) support this notion. Collectively, these findings indicate that the inactivation requires contact between protozoa and virus.

### T. pyriformis captured A(H1N1)pdm09 viruses into endosomes of two types

**Fig. 4:**
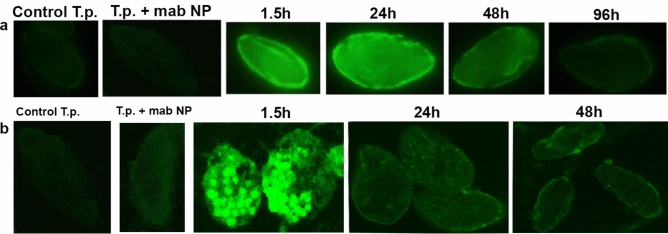
Temporal and spatial distribution of A(H1N1)pdm09 captured by *T. pyriformis* cells. The A(H1N1)pdm09 virus and *T. pyriformis* were co-cultivated at 25°C; at specified time points 100 μl samples were transferred to wells of the 96-well plates/glass slides, fixed with paraformaldehyde and A(H1N1)pdm09 was visualized using nucleoprotein -specific monoclonal antibodies (mabs NP). The images were taken with fluorescent (a) and CLSM (b) microscopy. A comparison is provided as a control the staining of control protozoan cells with those treated with the mabs to NP.

To understand mechanisms of A(H1N1)pdm09 inactivation by *T. pyriformis* cells, we used microscopy with capsid specific antibodies (Fig. 4). The fluorescent microscopy affirmed that virus was captured by protozoan cells (Fig. 4a). The efficiency of staining decreased with time to disappear 96 hpi that was in line with the data of RT/Q-PCR and HA assays. The confocal laser scanning microscopy (CLSM) was used to analyze intracellular virus distribution (Fig. 4b). CLSM revealed multiple brightly colored phagosomes within *T. pyriformis* cells 1.5 hpi. Twenty-four hours h later, the cytoplasm of protozoa weakly fluoresced, and against this background, phagosomes, which were much less bright than after 1.5 hours, were still noticeable. No visible phagosomes were observed within *T. pyriformis* cells 48 hpi although light fluorescence of the cytoplasm was still distinguishable.

### A(H1N1)pdm09 viruses associated with T. pyriformis cell lysates caused cytopathic effect in MDCK cells within first 48 h

**Fig. 5:**
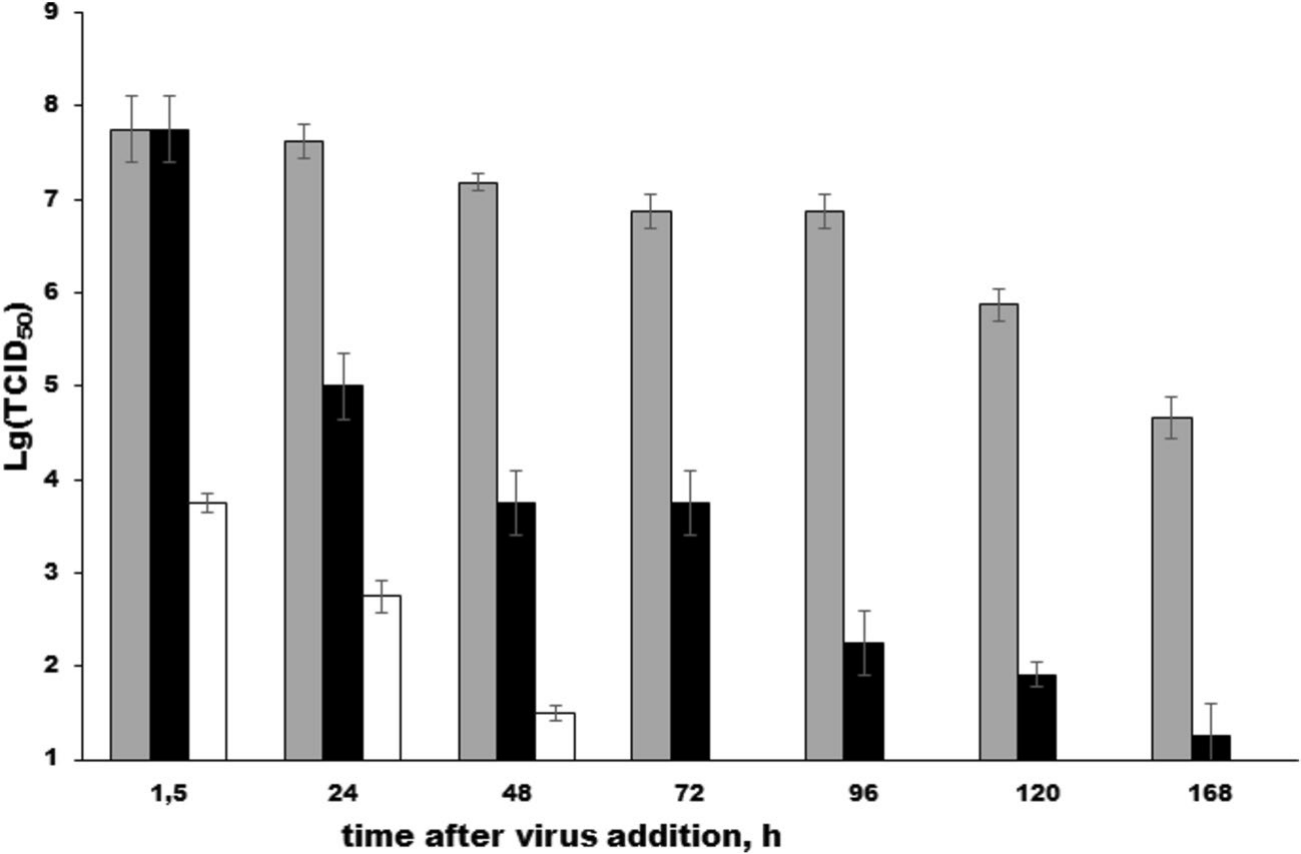
Cytotoxicity of the mixed A(H1N1)pdm09/T. pyriformis co-culture to MDCK cells. The TCID_50_ assay was performed on MDCK cells infected with lysates (white) or cell-free culture supernatant (black) of A(H1N1)pdm09-infected *T. pyriformis* cells. Lysates were made at specified time points after *T. pyriformis* infection with (H1N1)pdm2009. The pure A(H1N1)pdm09 culture was used as a control (grey). The mean values and Standard Deviation (SD) from 3 independent experiments are shown, *p*<0.05

The presence of visibly intact viruses within protozoan phagosomes suggested that at least a part of the associated with protozoa viral population was undamaged and potentially virulent within the initial hours of interaction. To verify this suggestion, we infected MDCK cells with lysates of infected *T. pyriformis* taken at different times points and with corresponding cell-free supernatants. Indeed, lysates from infected T. pyriformis produced cytopathic effects (CPE) in MDCK cells within first 24 h. The CPE diminished to the threshold 48 hpi and it was not observed at later stages of co-incubation (Fig. 5). Correspondingly, the CPE in the cell-free supernatant gradually decreased to reach the threshold 120 hpi. Within the first 1.5 h, infectivity of virus in supernatant was similar to the control, while infectivity of virus in *T. pyriformis* lysates was at a substantially lower level. These findings align with TEM data and suggest that viral population associated with *T. pyriformis*- was rapidly inactivated, b while retaining some recoverable capacity. The TCID_50_ assay corroborated our assumption that a partial destruction of the virions took place to 168 hpi. The virion destruction resulted in decreasing of viral titers of intact viruses in the control culture and RNA destruction in the presence of the medium conditioned by *T. pyriformis* (compare Fig. 3a,b and Fig. 5).

**Fig. 6:**
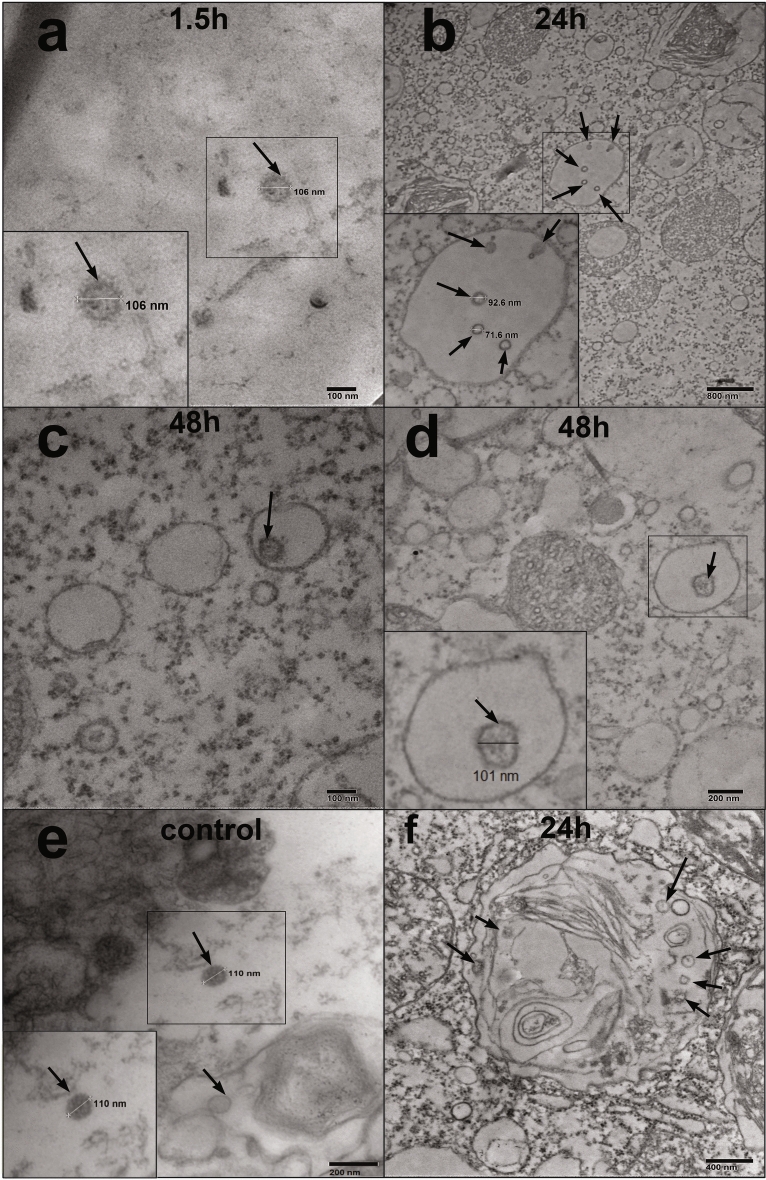
The TEM view of intracellular A(H1N1)pdm09 in *T. pyriformis* (a, b, c, d) and MDCK (e) cells. The A(H1N1)pdm09 virus and *T. pyriformis* were co-cultivated at 25°C. The samples of the A(H1N1)pdm09 / *T. pyriformis* co-culture were taken 1.5 h (**a**), 24 h (**b**, **f**), 48 h (**c**, **d**) post virus addition. For control of virus particles, virus-containing medium from MDCK cells infected with A(H1N1)pdm09 was utilized (**e**). Virus particles were observed in big (**a**, **b**, **f**) and small (**c**, **d**) membrane-coated endosomes. Small endosomes were coated predominantly (**c**) or half-smooth and half-coated (**d**). On the figure (**f**) bigger membranous structures were observed in both large and small phagolysosomes starting from 1.5 hpi. The virus particles are marked with arrows.

To get more evidences on interactions between protozoan cells and viruses, we performed transmission electron microscopy (TEM) of *T. pyriformis* from taken 1.5, 24, and 48 hpi. Across all time points, we found undamaged/partly damaged virus particles with the size of about 100-110 nm within membrane-coated endosomes (Fig. 6a,b,c,d,f). This size corresponded to viral particles observed within control MDCK cells (Fig. 6e). 1.5 and 24 hpi, large virion-including phagosomes were observed supporting results of CLSM microscopy (Fig. 6a, b). These phagosomes resembled food vacuoles formed in grazing *T. pyriformis*. 48 hpi, virions were found only within relatively small endosomes (Fig. 6c,d). Some of these virus-containing vesicles were half-smooth and half-coated (Fig. 6d) while others were predominantly coated (Fig. 6c), resembling the coated endosomes formed in MDCK cells infected influenza A. Round shaped particles with sizes ranged from 70 to 90 nm were identified as partially destroyed viruses lacking their surface proteins “brush” (hemagglutinin) (Fig. 6b,d), and bigger membranous structures were observed in both large and small phagolysosomes starting from 1.5 hpi (Fig. 6f). We suppose that the virus particles and intraphagosomal membranous structures might represent different stages of virus digestion. This hypothesis is consistent with the HA-test data, and the varying mechanisms of viral penetration over time. Up to 1.5 hours, phagocytosis predominates; from 1.5 to 24 hours, there is a decrease in phagocytosis; and from 24 hours onward, endocytosis predominates (Figs. 4 and 6).

**Fig. 7:**
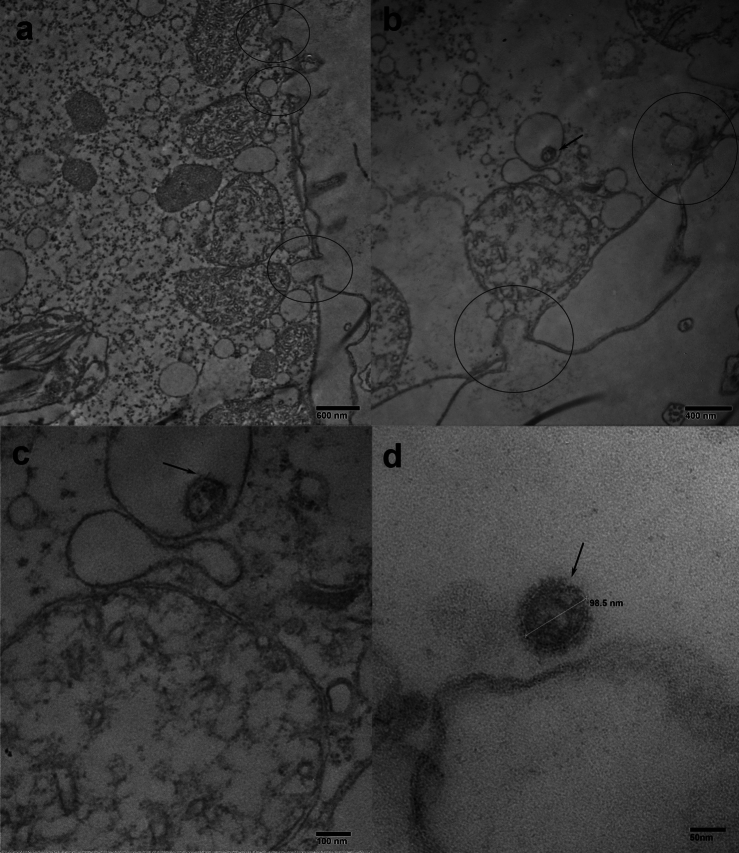
The TEM view of formation of small endosome (**a**, **b**, **c**) through of parasomal sacs / pellicular pores (**a**, **b**) in the cytoplasm at 48 h post-infection. Intracellular undamaged virion A(H1N1)pdm09 is observed in small endosome of *T. pyriformis* (**b**, **c**). For control of virus particles (**d**), virus-containing medium from MDCK cells infected with A(H1N1)pdm09 was utilized. The viruses are shown by arrows, the formation of small endosome is marked with a circle.

The analysis illustrated the formation of small endosome (Fig. 7 a,b,c) originating from of parasomal sacs/pellicular pores (Fig. 7 a,b) within the cytoplasm at 48 hpi. Intracellular undamaged virion A(H1N1)pdm09 were detected within in small endosome of *T. pyriformis* (Fig. 7 b, c). These undamaged virion A(H1N1)pdm09 appeared similar to control virions (Fig. 7 d), indicating the persistence of virus particles for 48 hours and supporting our previous findings.

### Virus digestion in food vacuoles was accompanied by an increase in the number and activity of protozoan mitochondria

**Fig. 8:**
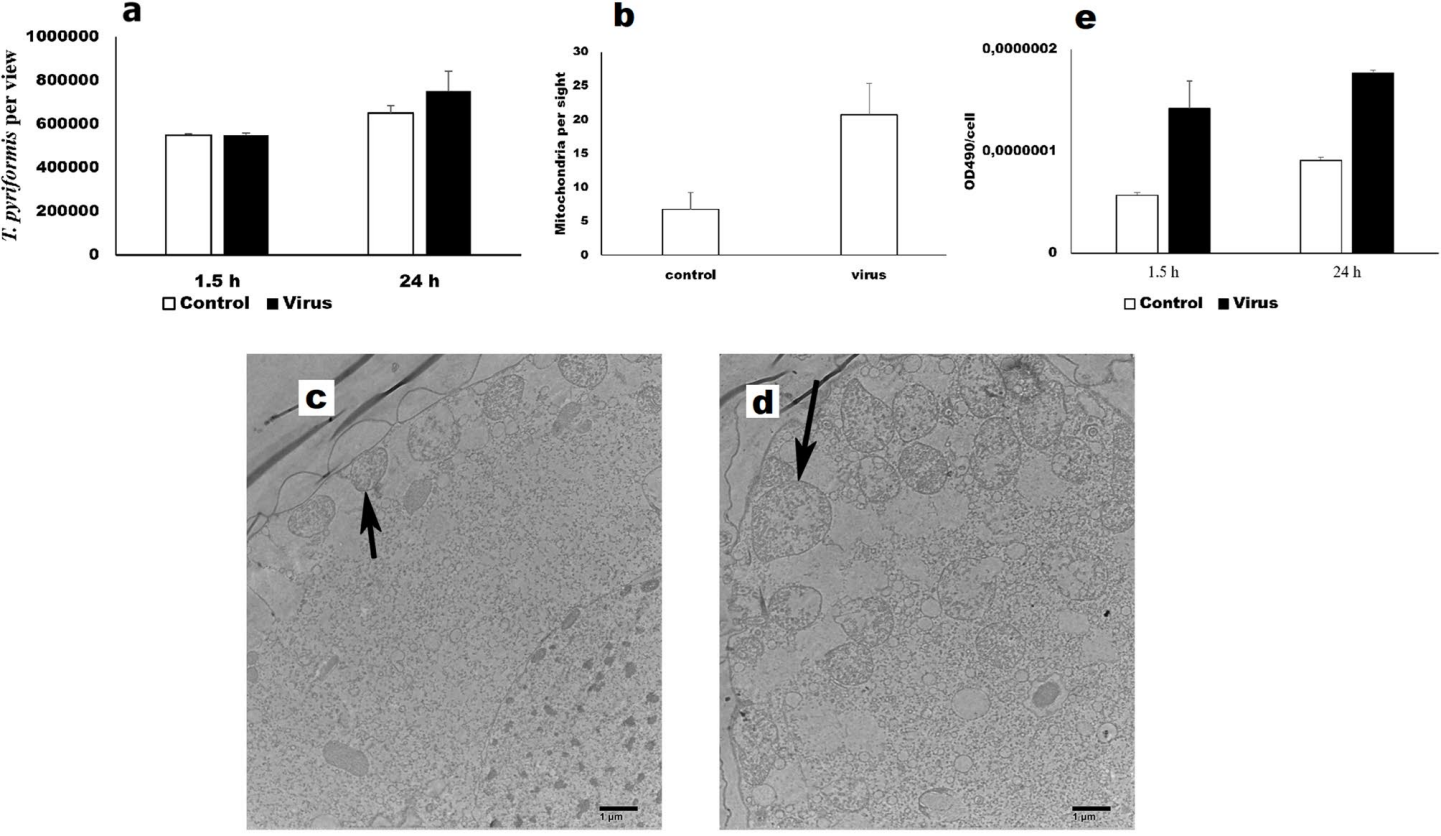
The metabolic activity of *T. pyriformis* co-culture with A(H1N1)pdm09 (virus) was analyzed using the MTT test (**e**). *T. pyriformis* was grown in Eagle’s MEM medium alone (control) or in the with A(H1N1)pdm09 (virus). Samples were collected 1.5 h and 24 h after virus addition. The number of T. pyriformis cells (**a**) was counted using light microscopy at magnification of 40X. The number of mitochondria (**b**) was counted using TEM: 1.5 h post addition of sterile medium (**c**) or virus (**d**), the mitochondria are shown with arrows.

Obtained data demonstrated that significant virus uptake and destruction occurred within first 24 h in parallel with active formation of big virus-containing phagosomes. To analyze the impacts of these activities on protozoan metabolism, we conducted the MTT test. Metabolic activities of protozoan cells increased 2.5-fold 1.5 post virus addition and about two-fold 24 h latter (Fig. 8e). The increase in metabolic activities correlated with the almost three-fold increase in the number of mitochondria 1.5 hours post virus addition (Fig. 8 b,c,d). These findings support the suggestion that the observed bid phagosomes represent food vacuoles forming by grazing protists. Despite this increased metabolic activity, introduction of viruses only slightly enhanced protozoan growth (Fig. 8a).

## Discussion

In this study, we demonstrated that prolonged up to 168 h co-cultivation of influenza A(H1N1)pdm09 and *T. pyriformis* resulted in a decrease and finally a total removal of the viral population. The inactivation of virus occurred due to endocytosis of viruses by protozoan cells but not due to effects of products excreted by *T. pyriformis*. Microscopic analyses revealed two types of virus-containing endosomes that dominated at different periods of interpopulation interactions and might differ in mechanisms of formation and virus processing.

We employed three methodologies, including the RT/Q-PCR, HA and TCID_50_ assay, to quantify the virus present in the protozoan cell lysates and culture supernatants. Although, these methods gave controversial results when analyzing viral dynamics, they provided varying insights into the internal mechanisms occurring within the protozoan cells and their culture media. HA test detected the activity of the surface viral protein hemagglutinin; while quantitative PCR quantified viral particle (viral RNA), and the TCID50 assay assessed the infectious activity of intact viral particles and the potential for damage restoration in compromised viral particles. Additionally, TEM Additionally, at the ultrastructural level. In the lysate of protozoan cells, the HA titer drops by 48 hours, which means that the HA protein is most likely inactivated by proteolytic enzymes inside the phagosomes of the ciliate, which is confirmed by confocal microscopy data.

The predominance of HA titers in the lysate relative to the supernatant, in contrast to the amount of viral RNA, is caused by a more active destruction of viral particles inside phagosomes than in the culture medium (the supernatant). Notably, in the viral control, titers initially decreased within 48 hours, followed by an increase at 96 and 168 hours as viral particle destruction and degradation intensified, even though infectivity (as measured by TCID50) was decreasing. Another possible explanation for the higher of HA titers in the lysate could be changes in the mechanisms of viral penetration into the *Tetrachymena*. Initially, phagocytosis through the cytopharynx predominated during the first 1.5 hours. Subsequently, from 1.5 to 24 hours, phagocytosis decreased, and after 24 hours, endocytosis became the dominant mechanism through parasomal sacs and pellicular pores in the cytoplasm. This observation correlates with phagocytosis dynamics observed through CLSM and aligns with the predominance of smaller endosomes as indicated by TEM data.

The RT/Q-PCR assay revealed viral RNA up to 96 hpi in *T. pyriformis* lysates and up to 168 hpi in the culture supernatant. The HA assay indicated a stable viral population in both cell lysates and culture supernatants up to 48 h, after which a decline led to eventual disappearance by 72 hours into the experiment. The TCID_50_ assay suggested that viruses associated with the protozoa retained their virulence for 48 h only. The results from the infectivity test imply that that the HA assay may detect partly destroyed surface viral proteins and virions, while, the RT/Q-PCR likely reveals viral-specific RNA from destroyed virions. The relatively long detection time of A(H1N1)pdm09 detection by RT/Q-PCR can be attributed to high sensitivity of the method. Collectively, these findings indicate relatively rapid inactivation of Influenza A(H1N1)pdm09 virus uptaken by *T. pyriformis*.

The rapid inactivation of A(H1N1)pdm09 suggested its heightened sensitivity to digestion by ciliates compared to phages. The suggestion about the diverse virus resistance to digestion by protozoa is in line with observations by Olive et al., who recently demonstrated that water-borne viruses possess distinct resistance to removal by*T. pyriformis* ^35^. Battistini et al^5^ found adenovirus type specific immunofluorescence up to 105 days of adenovirus type 2 co-cultivation with another ciliate species *Euplotes octocarynatus*^5^. Similarly, Olive et al^34^ described the uptake of *Tetrahymena* from adenoviruses without inactivation, virus particles were detected outside the digestive vacuoles, within the cytoplasm. This phenomenon may elucidate the long-term preservation of adenoviruses within protozoa in aquatic ecosystems, as it protects the virus from degradation. Our results demonstrated that immunofluorescence and/or persistence of virus-specific RNA are not reliable markers for ensuring viral integrity. Moreover, it is probable that ciliate species might possess a different virus-destructive potential. However, TEM results indicating the presence of intact viral particles within small endosomes at the 48-hour mark suggest a brief retention of viral integrity within protozoa.

The microscopy studies demonstrated accumulation of viruses in protozoan endosomes suggesting that virus inactivation by *T. pyriformis* predominantly occurs within phagosomes/phagolysosomes. The rapid pH drop and the activation of proteolytic enzymes during phagosome maturation likely expedite the inactivation of enveloped influenza viruses compared to phages.

CLSM and TEM microscopy identified two types of virus-containing endosomes. The first was big phagosomes similar to food vacuoles formed by *T. pyriformis* and other bacteriovourus protists grazing on bacteria^3^,^40^,^44^, Aijaz et al^1^. Such big phagosomes were observed during the initial hours up to 24 hours following the introduction of the virus culture to the protozoa. By 48 hours post virus addition, TEM revealed the presence of small endosomes slightly larger than the viral particles. At this time point, CLSM registered discrete and markedly weaker fluorescence across the protozoan cell volume. The shift in the predominant type of endosomes corresponded with the dynamics of the intracellular accumulation of A(H1N1)pdm09 as it was demonstrated by the HA test. This correlation suggests potential differences in the quantity, efficiency, and mechanisms of endosome formation of ”big” and “small” endosomes.

The formation of big virus-containing food vacuoles has been documented in *Tetrahymena* spp with *E. coli* phages. *E. octocarynatus* forms similar food vacuoles grazing on the adenovirus^5^. Viruses and phages are smaller than particles that trigger phagocytosis in ciliates, which are typically above 0.2 μm^44^. Pinheiro et al^38^ suggested that virus uptake by *Tetrahymena* may occur via macropinocytosis through the cytopharynx of the oral apparatus^38^. Pinocytosis is a process of fluid internalization. Macropinocytosis involves fluid internalization, enabling the formation of food vacuoles containing culture fluid when *Tetrahymena* grown in fluid axenic medium lacking any particles^43,44^. Prolonged cultivation of ciliates in such a medium as well as starvation immediately before addition of the viral culture might be a trigger to induce macropinocytosis of the medium containing viruses^46^.The formation of A(H1N1)pdm09-containing food vacuoles was accompanied by up-regulation of protozoan cell metabolism that supports the notion of virus digestion and utilization. Nevertheless, virus utilization was not sufficient to provide a sustainable growth of the protozoan population thus confirming that viruses are not a preferable food source for *Tetrahymena*. Notably, there are reports about the ciliate *Halteria*, that reportedly feeds exclusively on chloroviruses, suggesting a unique phenomenon termed “virovory” (Sultana Q et al^11,51^, Vasuja P et al^55^).

Small one-virus endosomes observed at 48 hpi may represent an alternative uptake pathway that could operate in parallel or in the absence of food vacuoles. Among the known endocytic pathways *Tetrahymena*, the clathrin-dependent endocytosis seems to be the most relevant candidate for an uptake of individual virus particles. Ciliates form clathrin‐coated vesicles at the adjacent to cilia sites known as parasomal sacs where the plasma membrane makes direct contact with the cytoplasm^33^, Elde et al^32^. Mechanisms of clathrin‐coated vesicle formation have been under intensive studies in the last years (Elde^8,32,54^). However, signals that initiate clathrin-dependent phagocytosis in protozoa remain elusive. In epithelial cells the clathrin-mediated phagocytosis is an important internalization pathway for the Influenza A, which provides capsid uncoating and release of viral ribonucleoprotein complexes (vRNPs)^26,29,31,36,47^. Matlin et al^29^ observed both fully coated and half-coated and half-smooth virus-containing endosomes in the MCDK cells and suggested that clathrin-coated endosome lose their coating upon fusing with other vacuoles^29^. Our observations of similar half-coated as well as fully coated virus-containing endosomes in *T. pyriformis* cells underscore the potential role of clathrin-mediated phagocytosis in viral uptake by free-living protozoa.

In summary, the results obtained in this study demonstrated that the interactions between Influenza A(H1N1)pdm09 and *T. pyriformis* mainly result in inactivation of the viral population. The inactivation of A(H1N1)pdm09 by *T. pyriformis* occurs within endosomes and can be mediated by at least two distinct endocytic pathways thus providing a wider range of opportunities for the ingested virus. Significantly, the identification of two distinct types of virus-containing endosomes indicates varied pathways for viral uptake and processing. The initial phagocytic uptake transitioned to the formation of smaller endosomes, suggesting alternative internalization mechanisms such as clathrin-mediated endocytosis. These findings provide novel insights into the viral uptake strategies employed by T. pyriformis and underscores the potential ecological implications of protozoan interactions with viruses in aquatic ecosystems. Within limited timeframe of several hours following, infected *T. pyriformis* may provide a mechanism for the intact passage of A(H1N1)pdm09 virus along the food chain, a feature that could be particularly significant in rapidly changing conditions within natural water reservoirs, where a unicellular predator may quickly become prey for larger predators, including birds and mammals. Such dynamics may facilitate the transmission of intact viruses through the food chain, contributing to viral resilience in changing aquatic ecosystems.

## Data Availability

The authors declare that the data supporting the findings of this study are available within the paper. Should any raw data files be needed in another format they are available from the corresponding author upon reasonable request.
